# Editorial: Evolution of abiotic stress responses in land plants

**DOI:** 10.3389/fgene.2023.1321165

**Published:** 2023-11-14

**Authors:** Diaa Abd El Moneim, Ana Luísa Garcia-Oliveira, Mahmoud Magdy

**Affiliations:** ^1^ Department of Plant Production (Genetic Branch), Faculty of Environmental Agricultural Sciences, Arish University, El-Arish, Egypt; ^2^ International Maize and Wheat Improvement Center (CIMMYT), Nairobi, Kenya; ^3^ Department of Molecular Biology, College of Biotechnology, CCS Haryana Agricultural University, Hisar, Haryana, India; ^4^ Genetics Department, Faculty of Agriculture, Ain Shams University, Cairo, Egypt; ^5^ Plant Biology Department, Faculty of Biology, Murcia University, Murcia, Spain

**Keywords:** abiotic stress, land plants, molecular responses, plant stress biology, climatic changes

## Introduction

The Genomics of Plants and the Phytoecosystem section at Frontiers in Genetics mainly publishes applied studies examining how plants can be improved using modern genetic techniques and tools. This Research Topic was designed to allow the publishing of new and upfront research related to plant stress biology and its coping mechanisms, particularly at the genetic level. This is crucial as climatic changes are more pronounced nowadays and, in a context, where there is a continuous need to feed the growing population. No longer bound by previous limitations, the confluence of high-throughput technologies with decades of molecular research has brought new dimensions to our understanding of plant responses and tolerance mechanisms to abiotic stresses. From scorching heat to biting cold, from soil salinity to desiccation, land plants face multifaceted challenges. In this context, rapid technological advancements are essential to aid our understanding of their resilience and progress in our knowledge of how agricultural practices can become more sustainable.

This Research Topic was designed to understand different aspects of plant biology and genetics and to overcome diverse abiotic stresses. As our technological prowess expands from the genomic and transcriptomic to the metabolic scales, so does our insight into these intricate mechanisms of plant resilience. We stand at the cusp of discoveries that reshape our understanding: novel genes, innovative cis-element regulators, intricate post-transcriptional and post-translational regulations, and the complex dance of protein secretion and degradation.

In this matter, Zhao et al. transported us back to the dawn of land plants, examining the phospholipase D (PLD) gene family in the unexplored territories of mosses. It is well known that PLDs in plants are implicated in the resistance to abiotic and biotic stresses, but also in plant growth and seed development. The authors examined moss PLDs’ rapid evolutionary expansion and diverse functional adaptations driven by its evolutionary forces. The work highlights evolutionary selection events and intricate gene structures as a precursor to future functional studies, setting the stage for a deeper understanding of moss land colonization processes but also of PLD gene’s biological functions.

On the other hand, Contreras-Díaz et al. focused on Chañar plants (*Geoffroea decorticans*), to understand the resilience of this plant in the Atacama Desert’s extreme conditions. For the first time, the complete mitogenome of a plant from the genus Geoffroea was sequenced and assembled, allowing the understanding of positive selection for some genes, such as the *atp8* gene, during species evolution and consequent adaptation for this species under extreme climatic conditions. It is particularly important to find the retention of this plant’s sdh4, nad1, and nad4 genes and their correspondence in drought tolerance mechanisms. Many plants that grow under more favourable conditions have lost these genes and, thus, possessing lesser capability to cope with arid environments. Therefore, the understanding of the consequences and utility of the combination of conserved genes that facilitate drought stress is of great importance.

From the agricultural point of interest, peanuts (*Arachis hypongea L.*) are a major cash crop in many countries, contributing significantly to regions’ economies, including several African nations. They serve as an essential source of income for farmers and support livelihoods across the supply chain, but also a good source of nutrients and vitamins. Grown particularly in tropical areas, it suffers from the action of biotic and abiotic stresses. Conducting a pangenome-wide study, Fatima et al. demonstrated the preservation of structural features and evolutionary patterns within a species. As shown in mosses, in this study, positive and negative selection pressure also had implications at the gene duplication process. The application of the Random Forest machine learning model discerns the multi-faceted stress responsiveness of AhCRK genes, paving the way for its future application in enhancing crop genetics. By comprehensively analyzing gene structures, evolutionary patterns, and expression profiles, they’ve highlighted the CRK gene family’s vital role in bolstering the peanut against environmental challenges, especially under salt and drought conditions.

Other proteins, such as HSP70s proteins, have been previously shown that when overexpressed can increase heat and drought tolerance in model and crop plants. Studying reverse genetics of these proteins in watermelon (*Citrullus lanatus L.*), Wang et al., for the first time, characterized and analyzed the physical and chemical properties of the CLHSP70 protein family. The study found that watermelon ClHSP70s responses to ABA, drought, and cold stresses are tissue-specific related. These results provide a reference for a better understanding of this gene family in the crop and offer a roadmap for targeted crop improvement.

Many areas of the world are currently facing shifts in their optimal temperatures and humidity ranges, implying that crops must adapt to these changes and the changes imposed by pests and diseases. Genetic diversity is fundamental to drive plant adaptation and resilience, and its conservation and responsible management are critical factors for both natural ecosystems and human agriculture. In this matter, Bhardwaj et al. reviewed the existent genetic variability in mungbean (*Vigna radiata L.*) associated with heat tolerance. The study demonstrates the impact of heat stress on physiological and metabolic pathways, including morphological changes, such as failure of ovule fertilization, in plants under heat stress conditions. The authors emphasize the species’ adaptive responses, possible tolerance sources, and mitigation through agricultural practices. The study highlights the use of molecular markers, mutational breeding, and genome editing as tools to create variability for developing heat-tolerant mungbean genotypes.

## Perspective

This Research Topic delves into a multitude of facets within the realm of plant stress tolerance, spanning from practical showcases of adaptability to drought and heat stresses. It elucidates how genetic information can be crucial to unveiling new research paths and increasing Research Topic scope without excluding other essential disciplines such as botany and agronomy. Our aim is that these selected publications may provide advancement as well as enthusiasm in the plant scientific community to continuously grasp new knowledge and applications into practice.

